# Associations Between Hiccups and Subsequent Cancer Diagnoses: A Population‐Based Cohort Study

**DOI:** 10.1002/cam4.71441

**Published:** 2025-11-30

**Authors:** Filip Jansåker, Xinjun Li, Kristina Sundquist

**Affiliations:** ^1^ Center for Primary Health Care Research, Department of Clinical Sciences Lund University Malmö Sweden; ^2^ University Clinic Primary Care, Skåne University Hospital Malmö Sweden

**Keywords:** cancer, cohort, hiccough, hiccups, singultus

## Abstract

**Background:**

Several case reports have recounted hiccups as a possible symptom of cancer, but potential associations in large study samples have not been examined.

**Methods:**

This population‐based cohort study examined associations between hiccups and subsequent cancer diagnoses. Standardized incidence ratios (SIRs) and 95% confidence intervals (CIs) were calculated.

**Results:**

Of 8466 patients diagnosed with hiccups during the study period (1997–2018), 7531 (89.0%) were men, 4013 (47.4%) were diagnosed in primary healthcare, and 325 (3.8%) were diagnosed with cancer. In men, hiccups were associated with nervous system (SIR 1.94; 95% CI, 1.08–3.20), hematologic (1.52; 1.17–1.93), and gastrointestinal (1.61; 1.36–1.89) cancers, including esophageal (2.63; 1.47–4.43), stomach (2.27; 1.42–3.45), colon (1.61; 1.23–2.08), and liver (1.77; 1.05–2.80) cancers. In women, hiccups were associated with gastrointestinal cancer (2.23; 1.27–3.63). Particularly strong associations were observed within 12 months of follow‐up.

**Conclusion:**

These findings may be useful for clinical practice and could provide foundations for detailed clinical studies on hiccups as a potential clinical cancer marker.

## 1 Background

Several case reports have recounted hiccups as a possible presenting symptom of cancer [1, 2, 3, 4, 5, 6, 7], but potential associations in large follow‐up studies have not been examined. Although usually benign and self‐limiting, hiccups may sometimes harbor serious underlying etiologies that disrupt the hiccup reflex arc, causing severe, persistent hiccups that may prompt medical attention [8, 9, 10]. Previous research has shown that hiccups are common in patients with cancer and that cancer diagnoses are common among patients diagnosed with hiccups, particularly gastrointestinal cancers [11, 12]. We here present a population‐based cohort study, including primary healthcare data, examining potential associations between hiccups and subsequent cancer diagnoses.

## 2 Material and Methods

This is an open population‐based cohort study of patients diagnosed with hiccups in Sweden. Several national registers and primary healthcare data were used (see Supporting Information for further details). The 10th edition International Classification of Diseases (ICD‐10) diagnosis code (R06.6) was used to identify patients diagnosed with hiccups. The outcome was the subsequent first cancer diagnosis identified in the National Cancer Register, which includes ICD‐7 codes. Based on previous literature [1, 2, 3, 4, 5, 6, 7, 8, 9, 10, 11, 12], we included head and neck, gastrointestinal, thyroid, lung, nervous system, and hematologic cancers, with the specific ICD‐7 codes listed in Table S1. Baseline covariates were age, sex, calendar year, country of origin (born in or outside Sweden), education level (< 12 or ≥ 12 years), and region of residence (in or outside large cities). Missing data were observed for country of origin (1.7%), education level (21.4%; of which, > 50% were in patients aged < 25 years) and region of residence (10.6%), and were imputed in born in Sweden, < 12 years of education, and large cities, respectively.

Each patient was followed from the date (month, year) of the first hiccup diagnosis (starting on January 1, 1997) until the first subsequent cancer diagnosis, emigration, death, or end of study period (December 31, 2018), whichever came first. Standardized incidence ratios (SIRs) were calculated using indirect standardization methods, comparing the observed (O) incidence of subsequent cancer with the expected (E) incidence in those without hiccups in the total population. Calculations were standardized by age (10‐year‐age‐groups), sex, time period (5‐year‐period‐groups), and other covariates. Poisson distribution was assumed when calculating 95% confidence intervals (CIs) of the SIRs. No substantial overdispersion was observed, as the residual deviance was close to the degrees of freedom in the Poisson model (ratio = 0.9). Analyses were performed using SAS software v. 9.4 (SAS Institute Inc., Cary, NC, USA). The study was approved by the Ethical Review Board in Lund, Sweden. Use of secondary, nationwide data obtained from the Swedish authorities does not require informed consent.

## 3 Results

Table 1 shows that 8466 patients (89.0% men) were diagnosed with hiccups during the study period and before the potential cancer diagnosis. The incidence rate of hiccups was 2.62 per 100,000 person‐years (4.49 in men and 0.82 in women) and particularly high in older men, peaking at 27.21 in men aged 80–84 years (Figure S1). Most hiccup diagnoses occurred in primary healthcare (47.4%), followed by outpatient specialist care (32.5%) and inpatient (20.1%) settings (Table S2). The mean follow‐up was 17.9 years (standard deviation: ±6.7), during which 325 patients (3.8%) were diagnosed with cancer.

**TABLE 1:** Number of cases, mean age at diagnosis, and incidence rate of hiccup diagnosis in men and women (1997–2018).

	No. of patients diagnosed with hiccups	Mean age at diagnosis (±SD)	Incidence rate (per 100,000 person years)^a^	No. (%) of subsequent cancer diagnosis^b^
All	8466	60.5 ± 19.4	2.62	325 (3.8)
Men	7531	61.9 ± 18.0	4.49	294 (3.9)
Women	935	49.5 ± 25.6	0.82	31 (3.3)

^a^ Adjusted for World standardized population.

^b^ Including head and neck, thyroid, gastrointestinal, lung, nervous system, and hematologic cancers.

Table 2 shows elevated SIRs for most cancers in men following hiccups, except for anorectal cancer. The associations were significant for gastrointestinal (SIR 1.61; 95% CI, 1.36–1.89), nervous system (1.94; 1.08–3.20) and hematologic (1.52; 1.17–1.93), as well as specific gastrointestinal cancer subtypes—esophageal (2.63; 1.47–4.43), stomach (2.27; 1.42–3.45), colon (1.61; 1.23–2.08), and liver (1.77; 1.05–2.80) cancers. In women, hiccups were associated with gastrointestinal cancer (2.23; 1.27–3.63), specific gastrointestinal cancer subtypes, and head and neck cancer (3 events). In some cases, there were too few observations to obtain significant associations as well as to rule out an increased risk for some cancers.

**TABLE 2:** SIRs for subsequent cancer diagnosis in patients (*n* = 8466) diagnosed with hiccups, stratified by sex and follow‐up time from hiccups diagnosis.

Sex	Men (*n* = 7531)				Women (*n* = 935)			
Cancers	O	SIR	95% CI		O	SIR	95% CI	
Head and neck	14	1.31	0.72	2.21	3	**5.36**	**1.01**	**15.86**
Thyroid	2	1.29	0.12	4.75	2	4.35	0.41	15.99
Gastrointestinal	153	**1.61**	**1.36**	**1.89**	16	**2.23**	**1.27**	**3.63**
Esophageal	15	**2.63**	**1.47**	**4.34**	4	**20.00**	**5.20**	**51.72**
Stomach	22	**2.27**	**1.42**	**3.45**	1	1.92	0.00	11.02
Small intestine	6	2.60	0.93	5.69	2	**11.76**	**1.11**	**43.27**
Colon	60	**1.61**	**1.23**	**2.08**	8	**2.34**	**1.00**	**4.63**
Anorectal	18	0.88	0.52	1.40	0			
Liver	18	**1.77**	**1.05**	**2.80**	1	1.39	0.00	7.96
Pancreatic	14	1.45	0.79	2.44	0			
Lung	45	1.24	0.91	1.67	5	1.73	0.55	4.07
Nervous system	15	**1.94**	**1.08**	**3.20**	1	1.05	0.00	6.03
Hematologic	65	**1.52**	**1.17**	**1.93**	4	1.27	0.33	3.27
Hodgkin lymphoma	1	1.06	0.00	6.10	0			
Non‐Hodgkin lymphoma	25	1.49	0.97	2.21	0			
Myeloma	13	1.85	0.98	3.18	0			
Leukemia	26	1.43	0.93	2.10	4	2.94	0.77	7.61

Follow‐up time	≤ 12 months				> 12 months			
Cancers	O	SIR	95% CI		O	SIR	95% CI	
Head and neck	3	1.88	0.35	5.55	14	1.46	0.79	2.45
Thyroid	2	7.41	0.70	27.24	2	1.15	0.11	4.23
Gastrointestinal	77	**5.29**	**4.17**	**6.61**	92	1.05	0.85	1.29
Esophageal	11	**12.94**	**6.42**	**23.24**	8	1.58	0.68	3.13
Stomach	17	**11.04**	**6.42**	**17.71**	6	0.69	0.25	1.52
Small intestine	4	**11.43**	**2.97**	**29.55**	4	1.88	0.49	4.86
Colon	25	**4.39**	**2.84**	**6.48**	43	1.23	0.89	1.66
Anorectal	5	1.59	0.50	3.75	13	0.70	0.37	1.21
Liver	7	**4.67**	**1.85**	**9.67**	12	1.28	0.66	2.24
Pancreatic	8	**5.41**	**2.31**	**10.70**	6	0.66	0.24	1.45
Lung	17	**3.03**	**1.76**	**4.86**	33	0.99	0.68	1.39
Nervous system	4	3.23	0.84	8.34	12	1.61	0.83	2.82
Hematologic	20	**3.13**	**1.91**	**4.83**	49	1.24	0.91	1.64
Hodgkin lymphoma	1	6.67	0.00	38.22	0			
Non‐Hodgkin lymphoma	9	**3.60**	**1.63**	**6.86**	16	1.04	0.59	1.69
Myeloma	5	**4.85**	**1.53**	**11.42**	8	1.24	0.53	2.46
Leukemia	5	1.84	0.58	4.32	25	1.49	0.96	2.20

*Note:* Bold type: 95% confidence interval does not include 1.000.

Abbreviations: CI, confidence interval; O, observed; SIR, standardized incidence ratio.

For cancers diagnosed ≤ 12 months after hiccups, the SIRs were significantly elevated for gastrointestinal (5.29; 4.17–6.61), lung (3.03; 1.76–4.86), and hematologic (3.13; 1.19–4.83) cancers, with particularly high SIRs for esophageal (12.94; 6.42–23.24) and stomach (11.04; 6.42–17.71) cancers. For cancers diagnosed > 12 months after hiccups, no significant associations were observed, though the SIRs were slightly elevated for most cancers.

## 4 Discussion

The main findings from this population‐based study show that hiccups are primarily diagnosed in men and in primary healthcare settings, and succeeded by cancer diagnosis in some (3.8%) patients. The majority of subsequent cancers occurred in men, who had significantly elevated incidence estimates of gastrointestinal (SIR 1.61), nervous system (SIR 1.94), and hematologic (SIR 1.52) cancers following hiccups. In women, hiccups were significantly associated with gastrointestinal cancer (SIR 2.23). Particularly strong associations were found for cancers diagnosed within 12 months after hiccups.

Previous research has shown that hiccups are common in patients with cancer [11] and vice versa [12], particularly gastrointestinal cancers. Several case reports have also described hiccups as a symptom of gastrointestinal [1, 2, 4, 6], nervous system [5, 7], and hematologic cancers [3, 4, 13]. The mechanism behind cancer leading to hiccups may involve disturbing neuroanatomic components of the hiccup reflex arc, directly or indirectly [8, 10]. For example, brain tumors may compromise the brainstem, and peripheral nerves (e.g., phrenic, vagal and sympathetic nerves) may be disturbed by cancers in the neck, abdomen, or thorax [1, 2, 3, 4, 5, 6, 7, 13]. Moreover, as vagal innervation of the gastrointestinal tract does not extend to the anorectal region [14], this may partly explain the associations between hiccups and gastrointestinal cancers (but not anorectal cancers). Additionally, gastrointestinal and lung cancers may cause hiccups by direct irritation of the diaphragma, and paraneoplastic syndromes might also contribute to hiccups (e.g., through electrolyte imbalances) [8].

The male dominance of hiccups observed in our study aligns with a systematic review [15], in which the authors suggest this may be due to higher excitability of peripheral nerves in the hiccup reflex arc among men. Additionally, biological and behavioral sex differences may contribute to higher cancer and disease burdens in men, which might lead to a higher hiccup risk.

The main limitation with our study is that the apparent underreporting of hiccups (probably mainly recorded when severe and persistent) may introduce selection bias and underestimate the true hiccup incidence in studies based on secondary register data. Although it is unclear how this potential bias may affect the results, it may represent a conservative bias, underestimating the strengths of the associations, or be differential, with hiccups being more common among patients with cancer [11]. Moreover, hiccups may represent a coincidental symptom unrelated to cancer, potentially leading to artifactual findings. In addition, patients with hiccups might also undergo diagnostic procedures (e.g., endoscopy, imaging) that may detect cancer to a higher degree than those without hiccups. These procedures could have been prompted by hiccups or other not accounted for factors—potentially resulting in residual confounding. Confounding from comorbidities (e.g., gastrointestinal and neurological disorders) and medications (e.g., systemic steroids, chemotherapy) that can contribute to both hiccups and cancer risk may also exist. As these potential confounders vary by cancer type, this is important to address in future studies on specific cancer types. Additionally, the absence of detailed clinical data prevented classification of hiccup diagnoses [8, 9, 10]. All this considered, detailed clinical studies are needed to explore potential clinical implications of our findings and clarify the role of hiccup presentation (e.g., severity and duration) in the identified associations. Nevertheless, the majority of hiccup diagnoses likely reflect severe cases prompting medical attention—e.g., persistent and intractable cases (known to harbor serious underlying etiologies)—rather than usual self‐limiting acute hiccups of low clinical significance [8, 9, 10].

There are also several strengths in this study that balance the limitations. First, it is a nationwide study, spanning over a long time period, with access to detailed individual‐level medical information from highly reliable sources with high completeness for the entire population and little loss to follow‐up. Second, the study includes data from almost nationwide primary healthcare settings, where most hiccups are diagnosed. Lastly, several of our findings were consistent with previous studies (e.g., mean age, male dominance, and low incidence rate of hiccups diagnosis) [12, 15], suggesting that our findings are robust and may be generalizable to patients diagnosed with hiccups in other healthcare settings. However, validation in other countries and populations is needed, given that the study is based on Swedish register data and differences in healthcare systems, diagnostic practices, genetic and environmental factors may limit its generalizability.

The present study expands previous case reports suggesting hiccups as a possible presenting symptom of cancer [1, 2, 3, 4, 5, 6, 7]. The findings indicate that the associations were confined to cancer diagnosed within 12 months of follow‐up, which suggests that hiccups are likely not an early clinical marker for cancer but might be a signal of co‐occurring cancer, potentially having prompted diagnostic investigations that led to cancer detection. Although hiccups have multiple causes, our findings indicate that clinicians should maintain an awareness of possible co‐occurring cancer in patients, especially older men and those seeking medical attention for severe, persistent hiccups, particularly when no other cause is evident, as some cancers may disrupt the components of the hiccup reflex arc [8, 10].

In conclusion, this population‐based cohort study identified potential associations between hiccups and certain cancers. These findings may be useful for clinical practice and could provide foundations for detailed clinical studies on hiccups as a potential clinical cancer marker.

## Author Contributions

Concept and idea: F.J. Development of idea and design: All authors. Funding and resources: K.S. and F.J. Access and acquisition of data: K.S. Analysis and statistics: X.L. with support from F.J. and K.S. Tables: X.L. and F.J. Interpretation of data: All authors. Literature search and drafting of manuscript: F.J. with support from X.L. Critical revision of the manuscript for intellectual content: K.S. and X.L. Guarantor of the study: K.S. All authors have approved the final version of the manuscript, attest that all listed authors meet the authorship criteria, and that no others meeting the criteria have been omitted. All authors take responsibility for the integrity of the accuracy of the data analysis, affirm that the manuscript is reported in an honest, accurate, and transparent account in regard to the study conducted and that no important aspects of the study have been omitted.

## Funding

The study was funded by non‐commercial research grants granted to Filip Jansåker by the Swedish Research Council (2024‐03748), Swedish governmental funding of clinical research (ALF‐2023‐YF0030), the Swedish Society of Medicine (SLS‐984649), and the Crafoord Foundation (20230633).

## Ethics Statement

The present study was a non‐intervention register study based on pseudonymized secondary data obtained from the Swedish authorities and was approved by the Ethical Review Board in Lund, Sweden (Dnr. 2012/795 and later approved amendments). The use of secondary, nationwide data obtained from the Swedish authorities does not require informed consent.

## Conflicts of Interest

The authors declare no conflicts of interest.

## Supporting information


**Data S1:** cam471441‐sup‐0001‐Supinfo.docx.

## Data Availability

This study made use of national registers and, owing to legal concerns, data cannot be made openly available. Further information regarding the health registries is available from the Swedish National Board of Health and Welfare (https://www.socialstyrelsen.se/en/statistics‐and‐data/registers/) and from Statistics Sweden (https://www.scb.se/en/).
