# Supplementary material for: Associations Between Hiccups and Subsequent Cancer Diagnoses: A Population‐Based Cohort Study
Source: Cancer Med. 2025 Nov 30;14(23):e71441. doi: 10.1002/cam4.71441 (PMC12665188; doi:10.1002/cam4.71441)

**Supplementary material**

**Supplementary information:** Description of the data sources used in the study

*Page 2.*

**Table S1.** Cancer diagnosis codes

*Page 3.*

**Table S2.** Number of hiccup diagnosis (ICD-10 diagnosis code: R06.6) by setting

*Page 4.*

**Figure S1.** Incidence rates (95% confidence interval in error bars) of hiccup diagnosis per 100,000 person-years (y-axis) in Sweden, by age (years) at the time of diagnosis (x-axis), 1997–2018

*Page 5.*

**Supplementary information:** Description of the data sources used in the study

*Population-based primary healthcare data* (1997–2018), which includes 10^th^ edition International Classification of Diseases (ICD-10) coded healthcare data from 20 out of 21 administrative healthcare regions in Sweden, was used in our study. The population-based coverage of this data varied over time and region throughout the study period, depending on the time of digitalization of the patient records in a certain region. In 2015, the register contained 72% of Sweden’s population and around 90% of the population at the end of the study period. For the current study, data on hiccup diagnosis was collected from these sources.

*The Swedish National Patient Register,* managed by the Swedish National Board of Health and Welfare (in Swedish: Socialstyrelsen), includes inpatient (1964–2018) and outpatient specialist care medical diagnoses (2001–2018). During the study period, all diagnoses were ICD-10 coded (1997–2018). For the current study, data on hiccup diagnosis was collected from this register.

*The National Cancer Register* (1958–2018, Socialstyrelsen), includes all cancer diagnoses (ICD-7 coded) in Sweden and was used for assessing cancer incidence and survival rates. Approximately 60,000 unique cancer cases are reported to this register annually. All cancer cases are attributed a date of clinical diagnosis. For the current study, outcome data was collected from this register.

*The Total Population Register* (1968–2018), which is managed by the Swedish governmental authority Statistics Sweden (SCB), includes data on the whole Swedish population, such as death, emigration, immigration, and sociodemographic characteristics. For the current study, data on country of origin, education level, and region of residence were collected from this register. Cancer incidence rates in the total population were calculated by combining data from the Total Population Register and National Cancer Register.

*Linkage of data:* At birth or upon immigration, all individuals residing in Sweden are assigned a unique 10-digit personal identification number. This number is utilized at all healthcare contacts and for the collection of data to national registers by the public authorities, thus enabling accurate linkage between medical data and public registries.

| **Table S1.** Cancer diagnosis codes | |
| --- | --- |
| Cancers | ICD-7 codes |
| Head and neck | 140, 141, 143–148, 161 |
| Thyroid | 194 |
| Gastrointestinal |  |
| Esophageal | 150 |
| Stomach | 151 |
| Small intestine | 152 |
| Colon | 153 |
| Anorectal | 154 |
| Liver | 155, 156 |
| Pancreatic | 157 |
| Lung | 162, 163 |
| Nervous system | 193 |
| Hematologic |  |
| Hodgkin lymphoma | 201 |
| Non-Hodgkin lymphoma | 200, 202 |
| Myeloma | 203 |
| Leukemia | 204–209 |
| ICD-7: The 7^th^ version of the International Classification of Diseases.  Data source: The Swedish National Cancer Register. | |

| **Table S2.** Number of hiccup diagnosis (ICD-10 diagnosis code R06.6) by setting | | | |
| --- | --- | --- | --- |
| Setting | No. | % | Mean age at diagnosis (± SD) |
| In-Patient^1^ | 1704 | 20.1 | 68.1 ± 15.1 |
| Out-Patient^1^ | 2749 | 32.5 | 57.8 ± 19.1 |
| Primary Health Care^2^ | 4013 | 47.4 | 59.1 ± 20.4 |
| All | 8466 | 100.0 | 60.5 ± 19.4 |
| ICD-10: The 10^th^ edition International Classification of Diseases. SD: Standard deviation. ^1^ National Patient Register. ^2^ Data from 20 out of 21 administrative healthcare regions. | | | |

**Figure S1.** Incidence rates (95% confidence interval in error bars) of hiccup diagnosis per 100,000 person-years (y-axis) in Sweden, by age (years) at the time of diagnosis (x-axis), 1997–2018


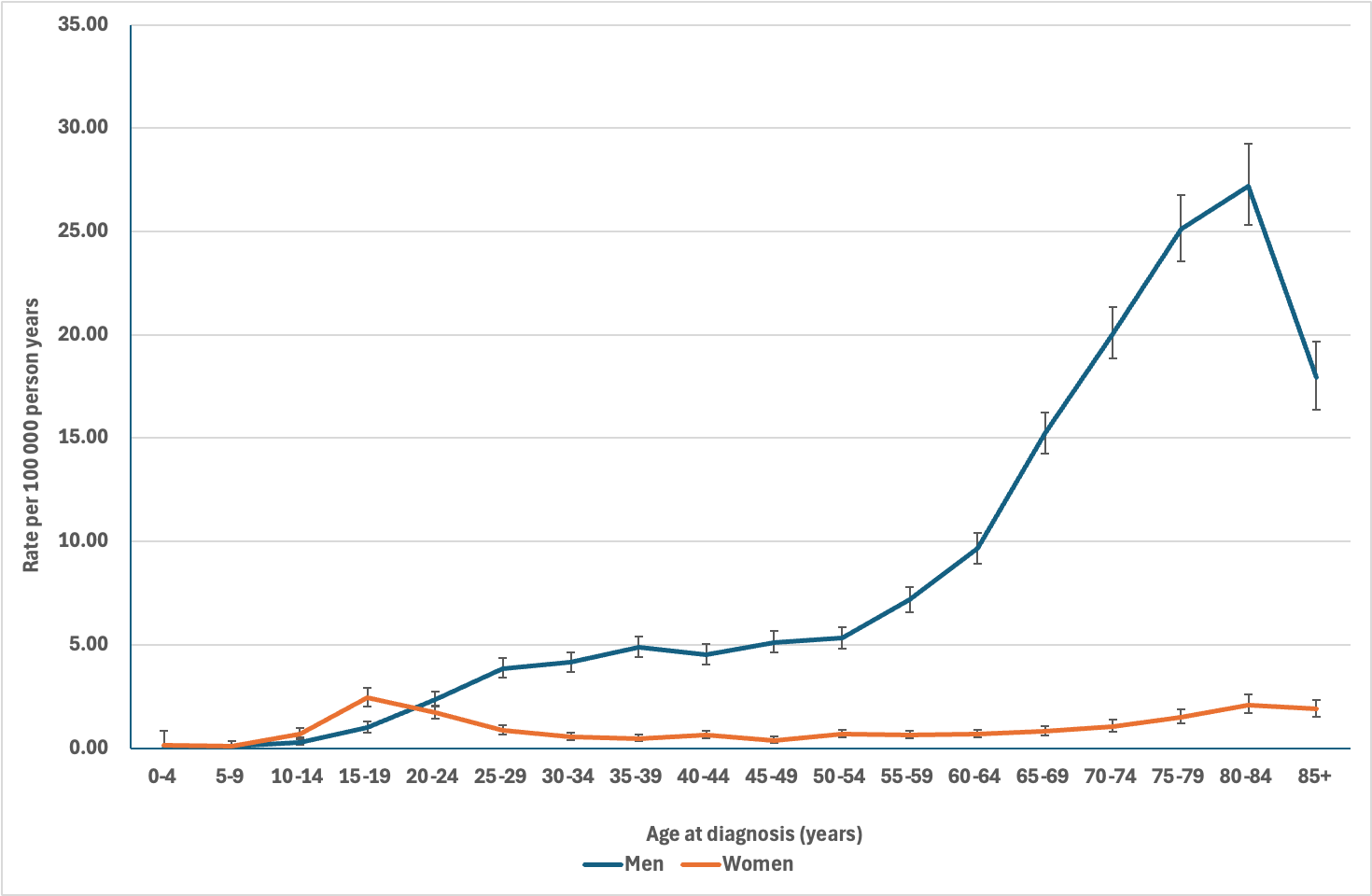

Supplement: Supplementary file 1 — Data S1: cam471441‐sup‐0001‐Supinfo.docx. [file CAM4-14-e71441-s001.docx]
